# Draft Genome Sequence of *Bacillus subtilis* GXA-28, a Thermophilic Strain with High Productivity of Poly-γ-Glutamic Acid

**DOI:** 10.1128/genomeA.01259-14

**Published:** 2014-12-04

**Authors:** Wei Zeng, Guiguang Chen, Zhen Tang, Hao Wu, Lin Shu, Zhiqun Liang

**Affiliations:** a State Key Laboratory for Conservation and Utilization of Subtropical Agro-Bioresources, Guangxi University, Guangxi, China; b Key Laboratory of Ministry of Education for Microbial and Plant Genetic Engineering, Guangxi University, Guangxi, China; c College of Life Science and Technology, Guangxi University, Guangxi, China

## Abstract

*Bacillus subtilis* GXA-28 is a thermophilic strain that can produce high yield and high molecular weight of poly-γ-glutamic acid under high temperature. Here, we report the draft genome sequence of this strain, which may provide the genomic basis for the high productivity of poly-γ-glutamic acid.

## GENOME ANNOUNCEMENT

*Bacillus subtilis* strain GXA-28 (CCTCC M2012347) is a Gram-positive, thermophilic, spore-forming bacterium with capability of high productivity of poly-γ-glutamic acid under high temperature, which was isolated from marine sands of Beihai, Guangxi, China (1). Poly-γ-glutamic acid or γ-PGA is a promising biomaterial with wide application in industry, agriculture, medicine, food, cosmetics, and wastewater treatment. Compared with the laboratory strain of *B. subtilis* Marburg 168 (2), the strain of GXA-28 can produce larger amounts of γ-PGA. The yield of γ-PGA, which was obtained at 45°C after 22 h, increased by 2.27 and 10 times over that obtained at 37°C and 28°C, respectively. Furthermore, the molecular weight reached 3.03 × 10^6^, which belongs to the range of ultra-high molecular weight (3). In order to explore the biosynthesis mechanism of high yield and high molecular weight of γ-PGA under high temperature, the genome of GXA-28 was sequenced and released.

The genome of GXA-28 was sequenced by using a shotgun strategy combining the Illumina HiSeq 2000 and Illumina MiSeq platforms, which produced paired reads totaling 1,260 Mb with 300-fold coverage. All of the sequence data were processed and *de novo* assembled into 13 contigs with an *N*_50_ of 1,100,410 bp, an *N*_90_ of 292,933 bp, and a maximum contig size of 1,156,606 bp using SPAdes version 3.0 (4). Annotation was conducted by RAST (5), RNAmmer (6), tRANScan (7), and BLAST against the RefSeq database. The genome of GXA-28 is 4,261,421 bp with a G + C content of 43.6%, containing 4,468 protein coding genes (CDSs), 71 tRNA genes, and 20 rRNA operons.

BLAST analysis of the genome sequence of GXA-28 against the RefSeq database revealed that it provided a complete set of genes related to γ-PGA biosynthesis, including the glutamate racemase genes *yrpC* and *racE*; the glutamate symport protein genes *gltT* and *gltP*; the synthetase genes *pgsBCAE*; the depolymerase genes *pgdS*, *ywrD*, and *ggt*; and the regulator genes *comPA*, *degSU*, *degQ*, and *swrA*. It has been reported that the two-component system (TCS) genes *comPA* and *degSU* were the key regulatory factors in the γ-PGA biosynthesis (8, 9). Interestingly, two temperature-responsive TCS genes, *vicKR* and *desKR*, which are involved in the regulation of the components of the cell wall/membrane and the desaturation of membrane phospholipids, respectively, are found in the genome of GXA-28. These may be associated with the high productivity of γ-PGA in GXA-28 under high temperature and provided novel information on the γ-PGA biosynthesis in *Bacillus* species, although much evidence is needed to verify this.

The availability of the genome sequence of a thermophilic strain GXA-28 provides us the opportunity to further understand the genetic differences between Marburg 168 and GXA-28 that affect γ-PGA biosynthesis, to explain the genetic reasons for high productivity of γ-PGA in GXA-28 under high temperature, and to get more genes that regulate or participate in the process of γ-PGA production.

### Nucleotide sequence accession numbers.

The whole-genome shotgun project has been deposited at DDBJ/EMBL/GenBank under the accession number JPNZ00000000. The version described in this paper is version JPNZ01000000.
